# Analysis of Spatial Offensive Performance in Handball: Differences between Men's and Women's Senior World Championships

**DOI:** 10.5114/jhk/170233

**Published:** 2023-10-11

**Authors:** Manuel Gómez-López, Jesús Rivilla-García, Iván González-García, Sergio Sánchez-López, Salvador Angosto

**Affiliations:** 1 Faculty of Sport Sciences, University of Murcia, Murcia, Spain.; 2 Faculty of Physical Activity and Sports Sciences, Polytechnic University of Madrid, Madrid, Spain.; 3 Faculty of Health Sciences, University Isabel I, Spain.; 4 Sport Research Group (CTS-1024), CERNEP, University of Almeria, Almería, Spain.

**Keywords:** match statistics, performance indicators, shooting, ranking, gender

## Abstract

The aim of this study was to carry out a descriptive analysis of the main performance variables of national teams that competed in the Men's (Germany-Denmark 2019) and Women's (Germany 2017) senior handball World Cups, and to compare the spatial offensive performance indices of laterality and depth according to the gender of players, considering for this purpose the total number of throws made according to the finishing area. A documentary study was carried out based on the total number of throws made in 192 male and 154 female games of 48 national teams belonging to 33 countries, which participated in previous World Cups. The data were collected from the International Handball Federation (IHF) statistics. The results showed that the areas from which the highest number of shots were taken in both World Cups were the central and shallow areas of the field. Several gender differences were observed. More specifically, male teams made much more attempts from the left side area than female teams (data), who finished from the right side (data). The depth index reflected that, although the dominant execution by gender was from deep offensive zones, men's teams finished more often from the 1^st^ offensive line, while female teams finished from the 2^nd^ offensive line. This information will be useful for coaches in designing training tasks and for players in improving decision making.

## Introduction

Currently, sports analysis has become a fundamental tool for investigating performance and the determinants of its success in team sports (O’Donoghue, 2015). Therefore, one of the most researched aspects in this context is the description of the competition and the determination of performance indicators through game analysis in order to collect objective, valid, accurate and reliable information (Antúnez et al., 2013; Blanco et al., 2015; Hughes and Bartlett, 2002; Hughes and Franks, 2007), that will help improve performance. This information will help coaches make management decisions, since it offers them a much more objective approximation of the reality of the game (García et al., 2011;Krawczyk et al., 2023; Ohnjec et al., 2008; Sargaet al., 2020; Trninic et al., 2010; Volossovitch et al., 2012). It will favour the design of specific training tasks, the development of competition strategies and the application of feedback to the athlete. In the same way, this information will also favour the improvement of the athlete's own decision-making (Escudero-Tena et al., 2020). Moreover, the ability to determine the relevance of different performance indicators can help establish collective strategies and tactics in sport(Petersen et al., 2008).

Although the benefits of sport performance analysis and evaluation are high, it is also complex to evaluate in the context of sport performance in team sports as it requires quantifying and qualifying the behaviours of the whole team (Blecharz et al., 2022;Garganta, 2007). In handball, for example, numerous variables have been used in different studies aimed at determining performance indicators that influence the final results of competitions (Antúnez et al., 2013; Gómez et al., 2014; Skarbalius et al., 2013; Vuleta et al., 2012). It should be stressed that the identification of these performance indicators in team sports is quite complex because of the speed of the game, constant changes of ball possession and phases of the game, the oppositional component and the difficulty of measuring the heterogeneity of elements that interact to achieve sporting success (Blecharz et al., 2022;Daza et al., 2017; Krawczyk, 2020; Milanović et al., 2018; Russomanno et al., 2021).

Finally, it should be noted that González (2019), based on a review of the literature, classified performance indicators into four types: (i) action variables, which include shooting actions (i.e., shooting efficiency), offensive actions (i.e., assists, technical fouls, turnovers or fouls reflected in the rules such as passing, doubles, area invasion or fouls in attack) and defensive actions (i.e., blocks, recoveries, interruptions originated by defensive behaviour such as free hits and exclusions or disciplinary sanctions); (ii) spatial variables, which consider finishing distances that each team uses in a particular situation, i.e., the majority areas of the pitch where teams finish their attacks (García et al., 2004, 2006); (iii) situational variables, based on the principle of obtaining success through a numerical advantage in a given area of the field; and (iv) temporal variables, due to the fact that time is a structural element closely related to space, since all actions take place in a given spatio-temporal sequence. These variables include offensive efficiency according to the periods of play and according to the duration of attacks together with the analysis of timeouts.

Focusing on the spatial variable as an index of offensive performance, it should be pointed out that this index allows us to visually obtain the most effective areas of a team and the volume of finishing in each attacking area. In this way, differences can be established between the most effective areas of teams and the degree of depth (effective distance of play) and width (effective side of the field of play) in terms of their finishing (González, 2019). It should be noted that so far, there have been few studies that analyzed the finishing zone as a performance factor and its influence on the effectiveness of teams during competition (López-León, 1999; Román, 1998), differentiating between the depth and laterality of different finishing zones and even more so considering the gender variable.

Given the above, the aim of the study was to carry out a descriptive analysis of the main performance variables of national teams that competed in the men's (Germany-Denmark 2019) and women's (Germany 2017) senior handball World Cups, comparing the finishing zones through the throws made and the laterality and depth indices of the men's and women's teams in both World Cups.

## Methods

### Participants

The International Handball Federation (IHF) organizes the Men's and Women's World Handball Championships every two years. The sample consisted of the total number of throws made in 192 men's and 154 women's games of 48 national teams belonging to 33 countries, which participated in both men's (Germany-Denmark 2019) and women's (Germany 2017) senior handball World Championships (Table 1).

**Table 1:** Number of matches played and the final position in the ranking of the participating teams in the men's and women's World Cups.

Country	Male		Female	
	Nº of participations	Ranking	Nº of participations	Ranking
Angola (ANG)	7	23	7	19
Argentina (ARG)	7	17	7	23
Austria (AUS)	7	19	-	-
Bahrein (BRN)	7	20	-	-
Brazil (BRA)	8	9	7	18
Cameroon (CMR)	-	-	7	20
China (CHN)	-	-	7	22
Croatia (CRO)	9	6	-	-
Czech Republic (CZE)	-	-	7	8
Denmark (DEN)	10	1	7	6
Egypt (EGY)	9	8	-	-
France (FRA)	10	3	9	1
Germany (GER)	10	4	6	12
Hungary (HUN)	8	10	5	15
Iceland (ICE)	8	11	-	-
Japan (JPN)	7	24	6	16
Korea (KOR)	7	22	6	13
Montenegro (MNE)	-	-	7	7
The Netherlands (NED)	-	-	9	3
North Macedonia (MKD)	7	15	-	-
Norway (NOR)	10	2	8	2
Paraguay (PAR)	-	-	7	21
Poland (POL)	-	-	7	17
Qatar (QAT)	7	13	-	-
Rumania (ROU)	-	-	6	10
Russia (RUS)	7	14	7	5
Saudi Arabia (KSA)	7	21	-	-
Serbia (SRB)	7	18	6	9
Slovenia (SLO)	-	-	6	14
Spain (ESP)	9	7	6	11
Sweden (SWE)	9	5	9	4
Tunisia (TUN)	8	12	7	24
Total	192		154	

### Measures

The variables used to observe the spatial offensive performance of the teams participating in the study were grouped as follows:

### Overall Match Information

The information collected from all the teams analyzed was as follows: the final position in the championship, the number of goals scored, the number of shots taken, total efficiency, the number of attacking possessions, the number of goals conceded, the number of total saves per game, the number of total shots conceded, defensive efficiency and opponent positions (Tables 2 and 3).

**Table 2:** Results of the variables analysed according to the team participating in the Men's World Championship.

Team	Ranking	Goals scored	Shots scored	Offensive efficiency	Possessions	Goals conceded	Saves	Shots conceded	Defensive efficiency	Possessions rival
		M(SD)	M(SD)	M(SD)	M(SD)	M(SD)	M(SD)	M(SD)	M(SD)	M(SD)
DEN	1	31.7(4.9)	46.7(4.9)	63.7(6.0)	49.8(5.5)	22.3(4.2)	12.7(3.6)	35.0(4.5)	36.2(9.4)	50.0(5.9)
NOR	2	32.5(5.8)	47.5(2.8)	61.0(7.5)	53.2(4.6)	25.6(3.6)	12.4(4.8)	38.0(3.4)	32.2(10.6)	52.8(4.2)
FRA	3	27.8(4.8)	44.5(5.6)	54.9(8.2)	50.7(4.2)	25.1(5.2)	11.5(4.1)	36.6 84.0)	31.5(10.37)	50.5(4.6)
GER	4	26.9(4.2)	41.9(6.6)	56.1(5.9)	47.8(4.4)	23.7(4.2)	10.5(3.4)	35.0(3.9)	30.2(9.4)	48.0(4.1)
SWE	5	30.3(4.8)	47.0(5.1)	57.7(7.3)	52.4(2.6)	24.7(5.2)	14.0(3.21)	38.7(5.4)	36.5(8.6)	52.6(2.3)
CRO	6	27.8(4.8)	42.3(3.0)	55.2(7.2)	50.1(4.8)	24.4(5.1)	12.4(3.4)	36.9(6.2)	33.8(6.6)	50.0(5.3)
ESP	7	30.4(5.3)	47.4(4.0)	56.9(8.0)	53.3(4.7)	25.9(4.5)	12.1(3.0)	38.0(5.0)	31.9(6.9)	53.7(4.9)
EGY	8	26.8(4.6)	45.2(6.0)	52.4(5.4)	51.0(6.2)	27.8(4.7)	11.1(2.1)	38.9(5.7)	28.6(4.3)	50.9(6.1)
BRA	9	26.5(5.0)	46.0(3.3)	48.5(7.8)	54.5(3.4)	27.5(5.1)	11.3(2.6)	38.8(4.7)	29.3(7.1)	54.1(3.4)
HUN	10	28.1(4.0)	43.6(4.9)	55.5(8.4)	50.9(2.8)	27.4(4.8)	11.5(3.1)	38.9(4.1)	29.7(8.3)	50.9(3.1)
ISL	11	25.9(5.1)	43.0(2.0)	52.7(11.2)	49.1(3.5)	26.4(5.7)	11.3(2.1)	37.6(5.4)	30.4(7.4)	49.4(3.3)
TUN	12	25.6(5.4)	45.4(5.6)	47.1(7.5)	54.3(3.8)	29.8(5.4)	11.1(3.5)	40.9(3.8)	27.5(9.3)	54.4(3.5)
QAT	13	28.0(5.6)	45.3(3.2)	54.4(10.1)	51.7(5.4)	26.0(3.3)	10.0(4.6)	36.0(4.0)	27.2(11.3)	51.3(5.5)
RUS	14	27.0(4.7)	45.3(3.0)	51.3(8.6)	52.9(5.3)	27.0(4.2)	12.3(4.1)	39.3(4.2)	31.2(8.9)	52.7(5.2)
MKD	15	27.3(6.2)	47.9(6.1)	50.0(10.0)	54.6(5.9)	28.4(3.5)	13.4(4.1)	41.9(6.3)	31.6(6.1)	54.4(6.4)
CHI	16	26.7(6.3)	47.0(2.0)	47.3(10.8)	56.6(1.6)	33.7(6.3)	9.3(3.8)	43.0(5.7)	21.8(8.7)	56.7(1.5)
ARG	17	24.7(5.7)	42.4(6.0)	49.0(12.0)	50.4(2.3)	25.7(3.2)	11.3(2.4)	37.0(2.8)	30.5(6.2)	50.7(2.1)
SRB	18	26.7(4.6)	44.6(3.5)	50.9(10.2)	53.0(3.3)	29.0(2.7)	9.7(3.3)	38.7(2.4)	24.9(7.7)	53.0(3.2)
AUT	19	24.6(4.3)	45.1(3.1)	47.0(8.3)	52.3(3.7)	28.4(4.5)	10.0(2.8)	38.4(3.3)	26.2(7.7)	52.9(4.1)
BRN	20	23.0(3.3)	43.0(4.6)	47.1(5.8)	48.7(2.0)	30.3(4.5)	9.0(3.5)	39.3(3.6)	22.9(9.1)	48.9(2.0)
KSA	21	24.7(5.0)	47.4(5.1)	44.0(8.0)	56.1(3.7)	30.6(5.3)	9.4(2.9)	40.0(4.9)	23.7(7.3)	55.9(4.1)
KOR	22	25.3(3.3)	45.3(4.5)	47.9(6.9)	53.0(3.6)	30.9(3.8)	10.7(4.7)	41.6(4.1)	25.4(9.5)	53.3(3.8)
ANG	23	26.0(4.2)	45.9(2.5)	46.4(8.1)	56.1(3.8)	31.9(4.6)	10.0(4.2)	41.9(1.7)	23.9(10.3)	55.7(4.1)
JPN	24	25.0(3.4)	45.4(5.3)	47.6(4.6)	52.7(5.9)	29.4(5.6)	7.9(3.4)	37.3(6.2)	21.0(7.6)	52.4(5.5)

**Table 3:** Results of the variables analysed according to the team participating in the Women's World Championship.

Team	Ranking	Goals scored	Shots scored	Offensive efficiency	Possessions	Goals conceded	Saves	Shots conceded	Defensive efficiency	Possessions rival
		M(SD)	M(SD)	M(SD)	M(SD)	M(SD)	M(SD)	M(SD)	M(SD)	M(SD)
FRA	1	26.2(3.8)	41.7(4.7)	49.8(5.4)	52.6(3.6)	21.0(4.1)	11.9(4.3)	32.9(2.6)	35.9(13.2)	52.2(3.0)
NOR	2	31.4(4.9)	47.7(7.0)	55.3(6.5)	56.6(5.0)	21.8(4.6)	14.4(6.2)	36.1(5.2)	38.8(14.7)	56.4(5.1)
NED	3	28.0(5.5)	48.2(4.7)	49.2(8.4)	56.8(3.0)	23.8(4.6)	11.6(4.6)	36.1(5.3)	31.8(11.3)	56.7(3.0)
SWE	4	29.1(6.0)	50.7(6.8)	49.8(6.3)	57.9(5.6)	25.7(4.3)	12.1(4.3)	37.8(4.5)	31.8(9.9)	57.9(5.7)
RUS	5	28.9(6.8)	46.1(4.4)	50.4(11.6)	57.3(4.1)	25.7(7.7)	11.9(3.3)	37.6(6.7)	32.5(11.0)	57.7(4.5)
DEN	6	26.6(5.9)	45.6(5.5)	49.7(9.7)	53.4(4.1)	23.3(6.3)	11.7(3.0)	35.0(5.4)	34.1(9.8)	53.1(3.8)
MNE	7	26.9(3.8)	46.7(4.4)	49.6(4.5)	53.9(4.1)	25.9(2.7)	9.1(3.2)	35.0(2.4)	25.9(8.0)	54.0(4.1)
CZE	8	26.9(5.1)	52.6(6.1)	45.1(8.2)	59.4(4.9)	29.1(4.9)	12.0(5.1)	41.1(8.4)	28.5(7.3)	59.3(5.1)
SRB	9	31.3(7.2)	48.7(5.6)	54.0(6.2)	57.5(7.5)	25.3(3.9)	12.0(4.9)	37.3(5.6)	31.6(9.8)	58.0(7.3)
ROU	10	25.0(5.7)	43.8(3.9)	49.2(10.5)	50.7(3.1)	23.3(5.1)	10.8(3.4)	34.2(3.3)	32.1(10.99	50.8(3.1)
ESP	11	26.3(6.0)	42.0(5.0)	49.3(8.8)	53.0(4.0)	23.3(5.6)	9.3(3.7)	32.7(3.1)	29.1(13.0)	52.3(3.6)
GER	12	22.8(3.5)	40.0(3.3)	46.8(8.4)	49.0(4.5)	19.3(7.4)	12.3(3.5)	31.7(6.7)	40.1(14.2)	48.8(4.1)
KOR	13	28.2(6.3)	46.8(10.2)	49.2(5.8)	56.8(8.6)	25.7(7.0)	11.3(3.4)	37.0(5.7)	31.3(10.7)	56.0(7.7)
SLO	14	26.5(3.8)	45.3(3.9)	47.5(8.7)	56.0(4.4)	27.8(5.1)	11.3(3.6)	39.2(2.6)	29.2(10.2)	56.7(4.0)
HUN	15	28.4(4.4)	45.0(4.8)	53.8(5.9)	52.6(5.9)	25.2(5.9)	11.6(2.5)	36.8(5.7)	32.1(9.5)	52.8(6.2)
JPN	16	26.3(4.7)	41.5(3.4)	46.5(9.1)	56.7(2.3)	25.0(6.8)	11.8(4.0)	37.8(3.4)	32.1(14.1)	56.8(2.4)
POL	17	29.6(6.0)	49.1(4.3)	48.1(9.0)	61.4(4.9)	29.3(4.9)	13.3(2.9)	42.6(3.0)	31.5(8.3)	61.3(4.6)
BRA	18	23.6(4.5)	42.7(4.6)	44.9(7.7)	52.7(4.2)	24.9(2.9)	10.9(1.9)	35.7(3.9)	30.4(3.64)	52.9(4.1)
ANG	19	27.1(5.5)	45.6(5.9)	49.7(7.4)	54.1(5.1)	28.3(3.7)	9.4(3.6)	37.7(3.3)	24.8(8.7)	54.3(5.3)
CMR	20	22.0(3.7)	44.9(5.5)	37.3(6.1)	59.1(5.7)	30.1(3.1)	11.9(5.7)	42.0(6.4)	27.2(10.1)	59.6(6.3)
PAR	21	20.9(6.0)	42.0(4.3)	36.6(11.5)	57.1(3.1)	28.9(4.7)	8.4(2.5)	37.3(3.4)	22.9(7.9)	57.7(2.9)
CHN	22	20.7(7.5)	44.0(3.5)	35.3(12.1)	58.1(6.6)	31.1(7.8)	9.6(4.2)	40.7(8.0)	23.5(9.7)	58.4(6.2)
ARG	23	22.3(4.4)	47.7(6.4)	37.3(6.8)	60.0(5.5)	31.4(6.9)	9.0(2.9)	40.4(7.8)	22.6(7.1)	60.0(5.8)
TUN	24	20.4(5.8)	44.9(6.1)	36.0(9.9)	56.7(2.8)	31.0(4.7)	10.1(3.1)	41.1(4.5)	24.7(7.1)	56.3(2.7)

### Shots Taken according to Areas of the Pitch and according to the Laterality and Depth of the Finishing Actions

**Figure 1:**
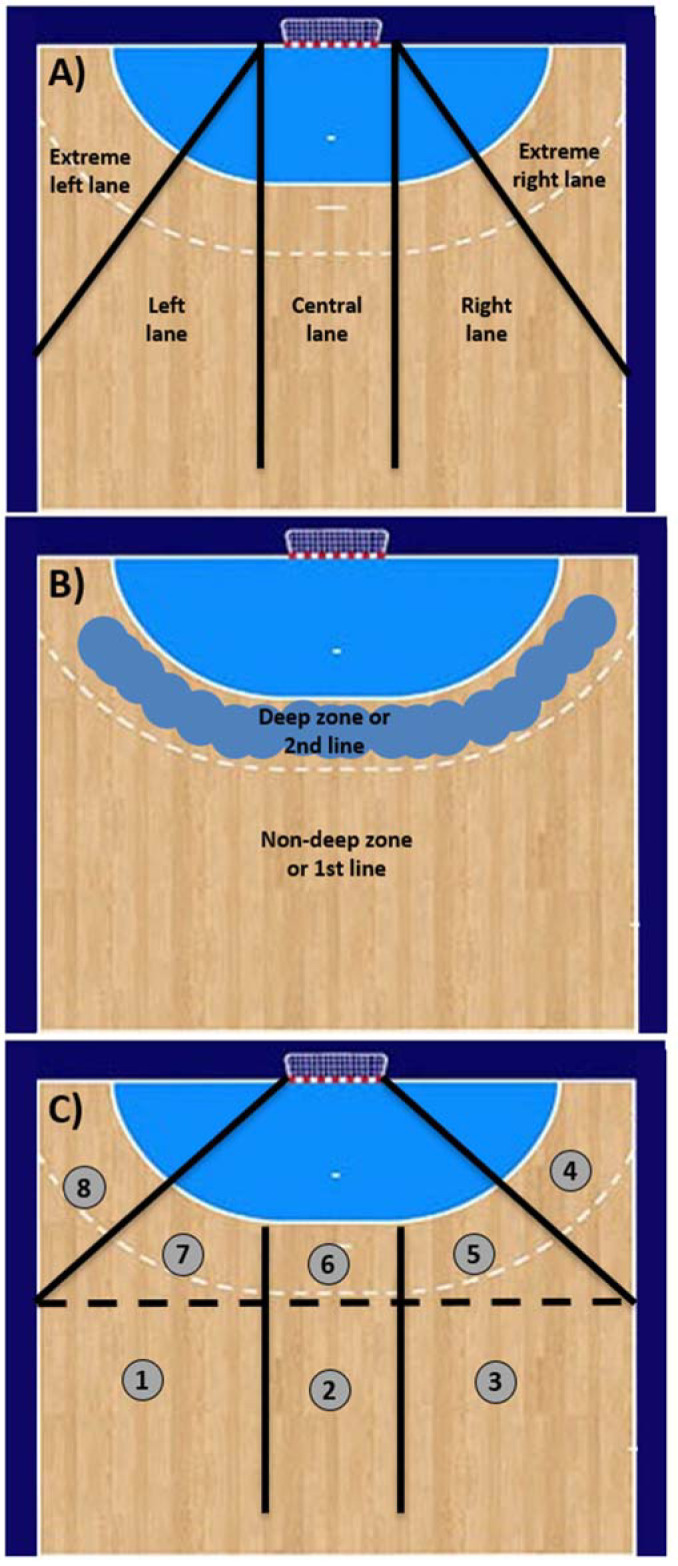
A) Zones depending on the laterality of the finishing actions. B) Zones according to the depth of finishing actions. C) Enumeration of zones according to laterality and depth of finishing actions.

The situation in which each throw was made was categorized according to laterality (Figure 1A) and the depth of finishing actions (Figure 1B), giving rise to eight throwing zones (Figure 1C) (González, 2019). The following are the different zones into which the offensive field is divided: the shallow left lateral zone (zone 1), the shallow central zone (zone 2), the shallow right lateral zone (zone 3), the far right zone (zone 4), the deep right lateral zone (zone 5), the deep central zone (zone 6), the deep left lateral zone (zone 7) and the far left zone (zone 8).

### Team Laterality Indices and Offensive Depth at the Spatial Level

According to previous research (González, 2019), the overall values of the Offensive Laterality Index (OLI) of each of the teams divided by each of the existing lanes on the field (left, central and right) were calculated together with the Offensive Depth Index (ODI), based on the distance of the shots with respect to the goal (deep zone and shallow zone). OLIs provide information about the effective side of the field of play and ODIs show the level of depth of the attack, giving information about the effective playing distance of the team or of any player. These indices were calculated following the indications of González (2019):

- Offensive Left Lateral Index (OLLI): Number of finishing actions on the left wing (zones 1 and 7) / Number of total finishing actions,
- Offensive Central Laterality Index (OCLI): Number of finishing actions in the centre (zones 2 and 6) / Number of total finishing actions,
- Offensive Right Lateral Index (ORLI): Number of completion actions on the left wing (zones 3 and 5) / Number of total completion actions,
- Offensive Depth Index at 1^st^ Line (ODI1): Number of completion actions at the 1^st^ line (zones 1, 2 and 3) / Number of total completion actions,
- Offensive Depth Index 2^nd^ Line (ODI2): Number of finishing actions at the 2^nd^ line (zones 4, 5, 6, 7 and 8) / Number of total finishing actions.

### Design and Procedures

First, all data pertaining to the XXVI Men's Handball World Championships held in Denmark and Germany in 2019, and XXIII Women's Handball World Championships held in Germany in 2017 that were available on the official website of the International Handball Federation (www.ihf.info) were collected. The instrument used for data collection was the official game statistics collection sheet. The data collectors were official statisticians of the competition who were previously trained to collect the data. These official statistics are a widely used instrument, especially in the field of sport. Likewise, in the scientific literature, it has been widely used in other team sports such as basketball with high reliability (Sampaio et al., 2004), while only few studies have been carried out in handball (García et al., 2008; Gómez-López et al., 2020; Ruiz-Sánchez et al., 2017; Sáez et al., 2009).

For the different World Cups, the squad sheets of each of the participating teams were downloaded, as well as the statistics sheets of all the matches of each of the championships. Once all the statistics spreadsheets were downloaded, a researcher recorded all the data between the 26^th^ of February and the 04^th^ of December, 2019 through an online form created by the 'Surveys' tool developed by the local university. The online form was used to ensure that the extraction of data from the match sheets was done correctly, no data were missed and there were no errors in recording data in different cells of a spreadsheet.

The following data were recorded: (i) goals scored and conceded; (ii) total and zone shots taken and conceded; (iii) offensive and defensive efficiency; (iv) team and opponent possession; and (v) total saves made. The data extracted were the number of shots taken that constituted an action with a completion of the ball towards the goal without the existence of an infringement during that action for each of the areas of the field according to their division by depth and laterality. Finally, the data were analysed and this report was written.

### Statistical Analysis

The data were analyzed using the statistical programme SPSS v24. For the continuous variables, mean and standard deviation were obtained. The normality of the variables was calculated using the K-S test. The U-Mann Whitney test for independent samples was also performed to observe the existence of differences in the statistics between the men's and women's World Cups and according to winning and last-place teams. The effect size was calculated using the rank biseral correlation (rbis), establishing a small effect for values of less than 0.10, a medium effect for values of 0.30, and a large effect for values of 0.50 (Coolican, 2009). The significance level was set at a value of *p* ≤ 0.05.

## Results

### Descriptive Analysis of Men's and Women's World Championships

Table 2 shows the results of the analysed variables of the different teams, ordered according to their final ranking, in the last men's World Championships in Germany-Denmark 2019, while Table 3 shows the results of the considered variables of the different teams, ordered according to their final ranking, in the last women's World Championships in Germany 2017. What stands out is the fact that, in both championships, offensive efficiency had a negative trend as the ranking was lower, i.e., in general terms the results were better in teams with a higher ranking. This was also the case for defensive efficiency, which was more significant in teams with higher rankings for both genders.

### Spatial Performance Indices according to Laterality and Depth of Finish of the Men's and Women's Teams

The results of spatial performance indices of the men's and women's teams participating in the last World Championships are shown in Table 4. Looking at the throwing zones, it can be observed that zones where the most throws were taken in both World Championships were central zones (Z2 and Z6). In the comparison between the men's and women's World Championships, it can be seen that men's teams performed a greater number of throws from Z2 (Men = 9.81 vs. Women = 8.24), Z6 (Men = 5.76 vs. Women = 5.31) and Z7 (Men = 2.23 vs. Women = 2.14), while women’s teams took more throws from Z4 (Women = 3.60 vs. Men = 2.86), Z5 (Women = 2.58 vs. Men = 2.42), and Z8 (Women = 3.51 vs. Men = 3.15). On the other hand, it should be noted that Z1 (Men = 3.51 vs. Women = 3.51) and Z3 (Men = 3.11 vs. Women = 3.11) had the same number of throws in both genders. According to the depth of throws made in the men's world championship, there was a balance between the throws from the first line (Z1, Z2 and Z3) and the second line (Z4, Z5, Z6, Z7 and Z8), with approximately 16 throws. Finally, we noted statistically significant differences in Z4 of the right winger and Z6 of the deep centre (*p*< 0.05). However, considering the size of the effect, Z2 or a non-deep central zone would also show significant differences in the number of throws between teams of both genders (rbis> 0.1).

**Table 4:** Results of the completion zones and the laterality and depth indices of the men's and women's teams.

Variables	Men's teams		Women's teams		U Mann-Whitney	*p* value	rbis
	M	SD	M	SD			
End zones							
Z1	3.51	2.0	3.51	2.6	15192	0.442	0.05
Z2	9.81	11.3	8.24	3.9	14321	0.097	0.10
Z3	3.11	2.0	3.11	2.1	15685	0.795	0.02
Z4*	2.86	1.9	3.60	2.1	12802	0.001	0.20
Z5	2.42	1.9	2.58	1.9	14796	0.235	0.07
Z6*	5.76	3.0	5.31	4.2	13748	0.024	0.14
Z7	2.23	1.7	2.14	1.8	15075	0.369	0.05
Z8	3.15	2.0	3.51	2.4	14798	0.239	0.07
Effectiveness of end zones							
Z1	42.5	31.2	36.0	31.1	12109	0.062	0.12
Z2	43.0	21.3	44.6	63.2	14496	0.163	0.09
Z3	38.3	32.6	40.2	33.5	13248	0.535	0.04
Z4*	59.4	35.5	51.9	30.4	119997	0.014	0.15
Z5	58.8	36.7	57.9	34.5	12226	0.707	0.02
Z6	65.5	24.5	69.9	35.8	14293	0.275	0.07
Z7	65.5	35.7	57.1	38.8	09921	0.068	0.12
Z8*	62.2	33.0	56.1	30.0	11950	0.034	0.13
Spatial Team Performance Indices – Laterality and Depth							
OLLI	0.127	0.054	0.123	0.064	15052	0.366	0.06
OCLI*	0.345	0.258	0.298	0.119	12985	0.003	0.19
ORLI	0.122	0.055	0.124	0.058	15624	0.750	0.02
ODI1	0.364	0.270	0.325	0.126	14504	0.143	0.09
ODI2*	0.443	0.099	0.474	0.132	13927	0.040	0.13

Note: * p < 0.05; rbis: rank biserial correlation: small effect (rbis > 0.10), medium effect for values of (rbis > 0.30) and large effect (rbis > 0.50)

In terms of efficacy, male players had higher efficacy in Z6 and Z7, both with 65.5% mean efficacy, while female players had higher efficacy in Z6 (M = 69.9%) and Z5 (M = 57.9%). Overall, males had higher efficacy than females in all zones except Z2 (Males = 43.0%; Females = 44.6%) and Z3 (Males = 38.3%; Females = 40.2%). Analyzing the results by world cup gender, there were significant differences in throwing efficiency in Z4 and Z8 (*p*< 0.05) although the effect of both variables was low. Both Z1 and Z7 had a high tendency to significance with low effect.

The results of team performance indices at the spatial level indicated that in relation to the laterality variable, in both championships the highest number of throws were made from the central zone (OCLI), showing symmetry between both lateral zones (OLLI and ORLI). However, differences were found between the men's and women's teams with respect to the incidence of laterality. Specifically, men's teams had their greatest participation in the left (Men = 0.127 vs. Women = 0.124) and the centre (Men = 0.345 vs. Women = 0.298) compared to women's teams that reached a greater incidence in the actions from the right (Women = 0.124 vs. Men = 0.122).

In terms of depth of shots, results showed that the highest number of shots were taken from the 2^nd^ line (Men = 0.443; Women = 0.474) compared to shots from the 1^st^ line in both the men's (2^nd^ line = 0.443 vs. 1^st^ line = 0.364) and women's (2^nd^ line = 0.474 vs. 1^st^ line = 0.325) World Cups. In addition, results reflected that men's teams finished more often from the 1^st^ line, while women's teams finished more often from the 2^nd^ line (ODI2). Finally, the OCLIs and ODI2 showed statistically significant differences between male and female teams (*p*< 0.05), with the effect of differences between these two variables being minor (rbis> 0.1).

## Discussion

The aim of the study was to perform a descriptive analysis of the main performance variables of national teams that competed in the men's (Germany-Denmark 2019) and women's (Germany 2017) senior handball World Cups, and to compare the spatial performance indices of laterality and depth according to the gender of players, considering for this purpose the total number of throws made according to the end zones. In accordance with the proposed goals, the results showed that both the men's and women's teams that participated in both World Cups made the greatest number of throws from central zones (Z2 and Z6), thus it was the central zone of the field that was most used to finish the game actions, which coincides with previous studies carried out in similar samples (Hatzimanouil, 2019; Jiménez-Olmedo et al., 2017; Ohnjec et al., 2008).

In the study of Hatzimanouil (2019), the larger number of throws were made from the central attack area with efficiency of 63.2%, to the left side of the goal at a low height. In fact, the efficacy of throwing from different attacking areas and player positions, from the same distance, exhibits heterogeneity. Similarly, in the study of Ohnjec et al. (2008), in the World Championship for women in Croatia 2003, the largest number of shots was taken from the backcourt positions with the shot efficacy of 34.10%, followed by the shots taken from the wings’ positions with efficacy of 50.07%, and shots taken from the goal area line with efficacy of 70.03%. In this study, the results reflected that the shot efficacy was better from the backcourt positions (around 40%) and wings’ positions (54%), while shots in the goal area were worst (around 62%). CherobiniPiovesan et al. (2020) analyzed different matches of the 2013, 2015, and 2017 women’s handball World Cups. The results of the previous study showed that the highest number of throws were made from the central zone. Specifically, while the Brazilian team shot mainly in the central region of the court (which presupposes great participation of the pivot), the French team showed a slight preference for the left region of the court, and the Norwegian team, on the other hand, presented a better balance between the right and left regions of the court (with greater participation of wing players).

The comparison of performance indices at the spatial level between both world championships according to laterality reflected that men's teams had their greatest participation on the left (Men = 0.127 vs. Women = 0.124) and the central wing (Men = 0.345 vs. Women = 0.298) compared to women's teams that obtained a greater incidence in actions from the right wing (Women = 0.124 vs. Men = 0.122). With respect to depth, the results showed that men's teams made a greater number of throws from shallower areas (1^st^ line) (Men = 0.364 vs. Women = 0.325), as opposed to women's teams who finished so with shots from deeper areas (2^nd^ line) (Men = 0.443 vs. Women = 0.474).

Similar results were previously obtained by García et al. (2004) with the top four teams of the ASOBAL league in the 2001–2002 season (Portland San Antonio, FC Barcelona, Ademar de León and BM Ciudad Real) and by Hatzimanouil (2019) with 25 high-level games of the Greek men's league. In the first of those studies, offensive finishing zones and the distance from which they were finished were differentiated. In this way, the following were determined as attacking finishing zones: two outer zones, two lateral zones and a central zone. In addition, they also analysed the distance from which the attack was finished, i.e., from 6 m, less than 7 m, between 7 and 9 m, and more than 9 m. The results obtained in both studies showed that the most effective areas were between 6 and 9 m. This study found that performance of throws and runs down the centre line was much more than twice as effective as those down the side lines (centre offensive line: around 3.3; side lanes: around 1.25). When considering the depth of the throws made, throws between 6 and 9 m were in the majority and represented higher effectiveness rates than long-distance throws where women were significantly more effective than men at shorter distances as discussed above. However, at long distances, men performed better, although the mean scores grouping depth indices are similar (around 0.40).

Similar results were also obtained by García et al. (2008) after recording the 2007 Spanish cadet men's championships. Although the aim of that study was to analyse counterattack goals and their shots, they confirmed that the deepest areas were the ones from which the greatest number of shots were taken. These studies reflect that there are different measurement variables depending on the study analyzed and that although players participating in both studies competed in national leagues, the results found in both world championships are similar.

Other studies with participants of the same sports level showed similar trends to those found in both world championships. Gheorghe and Mereuță (2020) analyzed matches of the women Romanian national team at the World Championships in Japan 2019, and found that the highest number of throws were made from the distances of 6 and 7 m, with the highest effectiveness from 7 m. Also, CherobiniPiovesan et al. (2020) showed that while the women's Brazilian team shot close to the opposing goalpost, the French team shot from long distances, while in the Norwegian team, there was a higher participation of wing players.

On the other hand, in the literature different results may be found, although with similar samples, to those found in both world championships analyzed, where the highest number of shots were made from the second offensive line. Meletakos et al. (2011) analyzed three consecutive men’s World Championships (2005, 2007 and 2009) and Meletakos et al. (2020) examined 300 matches played in the last eight World Men’s Handball Championships held in Tunisia 2005 (40 games), Germany 2007 (40 games), Croatia 2009 (40 games), Sweden 2011 (32 games), Spain 2013 (36 games), Qatar2015 (36 games), France 2017 (36 games), Germany/Denmark 2019 (40 games). In both studies it was indicated that the highest number of shots were taken from the distance of the first offensive line. Another study worth highlighting is the one carried out by Ferrari et al. (2020), which showed that the greatest number of goals were scored from the central, right wing and right winger areas.

Our results can have a great practical application as they offer a much more objective view of the reality of the game and allow the development of research on the analysis of sport competition. Furthermore, these results may be of help to coaches in the design of specific training tasks and in the development of competition strategies. Specifically considering the gender, women should focus on finishing from Z4 and Z8 as they have a greater number of final actions by wingers than men, however, their effectiveness was worse. Therefore, working to improve ball circulation and ball speed can lead to the creation of more space for female wingers to have a better shooting position and increase their effectiveness.

Nevertheless, further research is needed to fully understand the complex dynamics of offensive play in handball and to develop more refined and effective training strategies. Overall, this study represents a significant contribution to the ongoing dialogue around handball performance and may help inform future research and sports training.

Finally, some limitations of this study should be acknowledged as we did not analyze game actions that finished with a penalty throw and we did not include data from previous championships that would have allowed a longitudinal analysis of the men's and women's championships.

## Conclusions

This study shows that the areas from which the highest number of shots were taken in both World Cups were central and low depth zones. The comparison between World Cups indicates that men's teams finished, much more often than women's teams, from the centre and the left side and from shallower offensive zones, as opposed to women, who finished from the right wing and from deeper zones.

As a proposal for future studies from an offensive point of view, other indicators of team performance at a spatial level should be considered, such as the scoring rate and offensive spatial completion. In addition, the same performance indices could also be addressed from a defensive perspective.
